# Mid-term performance of His bundle pacing and usefulness of backup leads

**DOI:** 10.1093/europace/euae168

**Published:** 2024-06-14

**Authors:** Julia Herbert, Arianne Kovacsovics, Rita Brito, Nicolas Masson, Haran Burri

**Affiliations:** Cardiac Pacing Unit, Department of Cardiology, University Hospital of Geneva, Rue Gabrielle-Perret-Gentil 4, CH-1211 Geneva, Switzerland; Cardiac Pacing Unit, Department of Cardiology, University Hospital of Geneva, Rue Gabrielle-Perret-Gentil 4, CH-1211 Geneva, Switzerland; Cardiac Pacing Unit, Department of Cardiology, University Hospital of Geneva, Rue Gabrielle-Perret-Gentil 4, CH-1211 Geneva, Switzerland; Cardiac Pacing Unit, Department of Cardiology, University Hospital of Geneva, Rue Gabrielle-Perret-Gentil 4, CH-1211 Geneva, Switzerland; Cardiac Pacing Unit, Department of Cardiology, University Hospital of Geneva, Rue Gabrielle-Perret-Gentil 4, CH-1211 Geneva, Switzerland

**Keywords:** His bundle pacing, Conduction system pacing, Backup lead, Lead revision, Adverse events

## Abstract

Ventricular backup leads may be considered in selected patients with His bundle pacing (HBP), but it remains unknown to what extent this is useful. A total of 184 HBP patients were studied. At last follow-up, 147 (79.9%) patients retained His bundle capture at programmed output. His bundle pacing lead revision was performed in 5/36 (13.9%) patients without a backup lead and in 3/148 (2.0%) patients with a backup lead (*P* = 0.008). One patient without a backup lead had syncope due to atrial oversensing. Thus, implantation of ventricular backup leads may avoid lead revision and adverse events in selected HBP patients.

## Introduction

His bundle pacing (HBP) offers the most physiological form of cardiac stimulation, but is associated with elevated thresholds, sensing issues, and a high rate of lead revision.^1,2^ For these reasons, left bundle branch area pacing (LBBAP) has overtaken HBP in many centres.^3,4^ Nevertheless, there are advantages with HBP compared to LBBAP, such as more physiological ventricular activation, clear endpoints for conduction system capture, lack of tricuspid regurgitation,^5^ and extractability,^6^ which merit its continued use for conduction system pacing, either as a first-line approach or as rescue therapy in case of failed LBBAP.

Implantation of a ventricular backup lead in selected patients (e.g. pacemaker-dependent patients, infra-nodal block, ‘pace and ablate’, and sensing issues) is suggested in the 2021 European Society of Cardiology pacing guidelines (class IIa, level of evidence C).^7^ This strategy however results in more implanted hardware, increased cost, and complex programming.^8^ There are currently no data evaluating usefulness of backup leads in the setting of HBP.

The aim of this report is to review the mid-term retention of HBP and to evaluate the clinical impact of backup leads.

## Methods

The Geneva Conduction System Registry is a prospective registry that was approved by our institutional ethics committee. All patients gave written informed consent to participate. Consecutive patients with successful HBP implantation from May 2017 to June 2023 were included. An additional ventricular lead was implanted in case of (1) clinical indication, e.g. an implantable cardioverter defibrillator (ICD) lead or a coronary sinus lead for resynchronization therapy, (2) pacemaker-dependency, (3) ‘pace and ablate’ indication, or (4) R-wave sensing amplitude < 1.8 mV.

His bundle capture was confirmed by ECG criteria.^9^ Follow-up reports and ECGs recorded during threshold tests were analysed to evaluate loss of His bundle capture.

## Results

HIS bundle pacing was attempted in 222 patients and was successful in 191 (86%) cases. Follow-up was unavailable in seven patients, who were withdrawn from analysis. Demographics of the 184 included patients are summarized in *Table 1*. Median follow-up was 830 [IQR 238–1317] days, and 132 patients had >1 year follow-up.

**Table 1:** Patient demographics

	All (*n* = 184)	With backup lead (*n* = 148)	Without backup lead (*n* = 36)	*P*
Age (years)	74 ± 12	73 ± 13	78 ± 8	0.046
Male	105 (57)	82 (55)	23 (64)	0.45
Female	79 (43)	66 (45)	13 (36)	
Medical history				
Coronary angioplasty	85 (46)	76 (51)	9 (25)	0.53
Coronary artery bypass surgery	31 (17)	27 (18)	4 (11)	0.46
Dilated cardiomyopathy	11 (6)	11 (7)	0 (0)	0.13
Valve surgery	15 (8)	15 (10)	0 (0)	0.045
TAVI	5 (3)	4 (3)	1 (3)	1.0
Diabetes	50 (27)	39 (26)	11 (31)	0.68
Hypertension	107 (58)	85 (57)	22 (61)	0.71
Chronic renal insufficiency	45 (25)	38 (26)	7 (19)	0.52
Device indication				
Pace and ablate	55 (30)	55 (38)	0 (0)	<0.001
AVB I	7 (4)	6 (4)	1 (3)	
AVB II	37 (20)	22 (15)	15 (42)	
AVB III	41 (28)	29 (20)	12 (33)	
In lieu of BiVP-CRT	6 (3)	6 (4)	0 (0)	
HOT-CRT	22 (12)	22 (15)	0 (0)	
Syncope without documented arrhythmia	5 (3)	3 (2)	2 (6)	
Slow atrial fibrillation	9 (5)	4 (3)	5 (14)	
Painful LBBB	1 (1)	1 (1)	0 (0)	
Brady-tachy syndrome	1 (1)	0 (0)	1 (3)	
Intrinsic QRS				
Duration (ms)	117 ± 33	118 ± 33	110 ± 29	0.19
Normal	113 (61)	87 (59)	25 (69)	0.35
RBBB	38 (21)	32 (22)	7 (19)	
LBBB	27 (15)	24 (16)	2 (6)	
NIVCD/fascicular block	3 (2)	3 (2)	1 (3)	
Paced rhythm	3 (2)	2 (1)	1 (3)	
Implanted device				
CRT-P	78 (42)	78 (53)	0 (0)	<0.001
Dual-chamber pacemaker	81 (44)	46 (31)	35 (97)	
Single-chamber pacemaker	1 (1)	0 (0)	1 (3)	
CRT-D	24 (13)	24 (16)	0 (0)	
Implanted HBP lead				
3830	178 (97)	144 (97)	34 (94)	0.33
Solia	6 (3)	4 (3)	2 (6)	

Values are shown as mean ± SD and *n* (%). *P*-values calculated using Student’s *t*-test and Fisher’s exact test.

AVB, atrioventricular block; BiVP-CRT, biventricular paced cardiac resynchronization therapy; CRT-D, cardiac resynchronization therapy defibrillator; CRT-P, cardiac resynchronization pacemaker; HBP, His bundle pacing; HOT-CRT, His-optimized cardiac resynchronization therapy; LBBB, left bundle branch block; NIVCD, non-specific intraventricular conduction delay; RBBB, right bundle branch block; TAVI, transcatheter aortic valve implantation

Additional ventricular leads that served as backup were implanted in 148 (80.4%) patients. A right ventricular pacing lead was implanted solely for the purpose of backup in 97 (52.7%) patients. The backup lead was an implantable cardioverter (ICD) lead in 23 (12.5%) patients and a coronary sinus lead in 28 (15.2%) patients.

His capture threshold at implantation was 1.4 ± 0.9 V/0.6 ± 0.2 ms. Non-selective capture was present in 60 (32.6%) patients with an average myocardial capture threshold of 1.7 ± 1.7 V/0.5 ± 0.2 ms. Sensing amplitude was 3.2 ± 2.4 mV, and pacing impedance was 525 ± 141 Ω. Backup leads were implanted in 107/124 (86.3%) patients with selective capture and in 41/60 (68.3%) patients with non-selective capture (*P* = 0.006). At last follow-up, His capture threshold was 1.2 ± 0.5 V/0.6 ± 0.3 ms, sensing amplitude was 4.0 ± 1.7 mV, and pacing impedance was 360 ± 95 Ω (*P* = 0.001, *P* = 0.27, and *P* < 0.001, respectively, compared to implantation).

**Figure 1:**
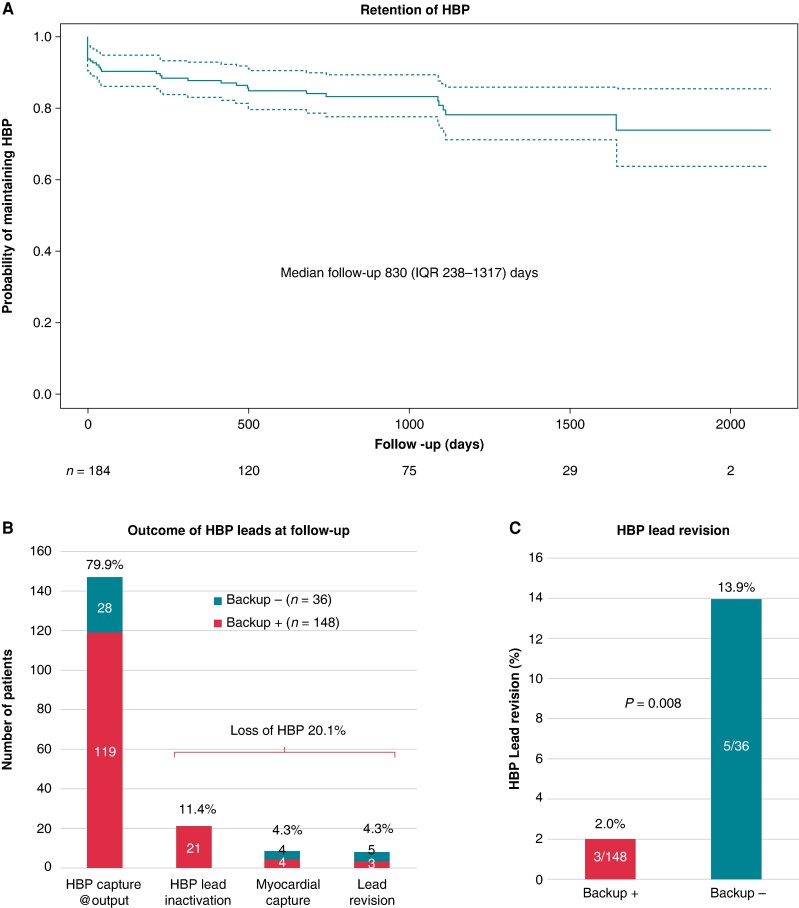
(*A*) Kaplan–Meier curves for retention of His bundle pacing (HBP) showing spontaneous loss of His capture throughout follow-up. (*B*) Outcome of the HBP lead at follow-up. Presence of His capture was evaluated at programmed pacing output (@output). (*C*) Incidence of lead revision in patients with and without backup leads (Backup+ and Backup−, respectively).

Retention of HBP at follow-up is summarized in *Figure 1*. A high HBP threshold (>2.5 V/0.4 ms or >2.0 V/1 ms) was present in 10/147 (6.8%) patients with retained HBP and required programming high pacing output. Iatrogenic loss of His capture occurred in 5/55 (9.1%) patients with a ‘pace and ablate’ indication (all had backup leads and none required lead revision).

A total of eight (4.3%) patients had HBP lead revisions due to elevated capture thresholds. His bundle pacing lead revisions occurred in 5/36 (13.9%) patients without and in 3/148 (2.0%) patients with a backup lead (*P* = 0.008). Patients with a backup lead had lead revision due to cardiac resynchronization therapy indication (*n* = 2) or due to painful left bundle branch block (*n* = 1). Patients were revised to LBBAP (*n* = 4), right pacing (*n* = 3), and to coronary sinus pacing (*n* = 1). Indications for pacing in the patients who underwent lead revision and who did not have a backup lead were slow atrial fibrillation (*n* = 1), second degree atrioventricular block (*n* = 3), and asymptomatic third degree atrioventricular block with an escape rhythm of 37 bpm (*n* = 1).

One patient with complete atrioventricular block without a backup lead had syncope four months after implantation due to atrial oversensing, which was solved by reprogramming sensitivity.

During follow-up, 37 (20.1%) patients died, of whom 33 had backup leads (*P* = 0.17 compared to no backup lead). Eleven patients died due to cardiovascular causes, 14 due to non-cardiovascular causes, and 12 patients had an unknown cause of death.

## Discussion

We report for the first time that patients with backup leads had a significantly lower requirement for lead revision compared to those without (2.0% vs. 13.9%, *P* = 0.008) due to alternative pacing modalities.

Loss of HBP occurred in 20.1% of our patients over ∼2 years, which is in agreement with previous studies that reported rates of up to 28%.^1,2^ Teigeler *et al*.^1^ report a 11.3% rate of lead revision that is higher than the 4.3% in our series. However, only 22% of their patients had backup leads, compared to 80% in our report. The many backup leads in our series are explained by the high proportion of ‘pace and ablate’ and high-degree atrioventricular block indications, and the requirement of additional ventricular leads for ICD or resynchronization therapy.

In addition to high capture thresholds, HBP may also result in sensing issues (as illustrated by one of our patients who had syncope due to atrial oversensing) for which a ventricular lead may provide more reliable values.

We currently only perform LBBAP in patients who require atrioventricular nodal ablation, as there is a significant risk of compromising HBP lead function in these patients (which was the case in 9.1% of our patients undergoing this procedure).

### Study limitations

Only a randomized trial may properly validate the impact of backup leads, but this will be difficult to conduct given high-risk pacing indications and the advent of LBBAP. This series included our initial learning curve and may yield different results with experience and evolution in HBP implantation technique.^10^

## Conclusions

Backup leads for HBP should be considered in selected patients who are at risk of asystole in case of loss of capture or of sensing issues, in the interest of safety and for reducing requirement for lead revision, which is demonstrated for the first time in this report.

## Data Availability

The authors will provide access to their data upon reasonable request.
